# HEALTH-RELATED QUALITY OF LIFE IN OLDER ADULTS AFTER A FLOOD

**DOI:** 10.1093/geroni/igad104.2666

**Published:** 2023-12-21

**Authors:** Piper Bordes, Katie Cherry, Matthew Calamia, Emily Elliott, Laura Sampson, Sandro Galea

**Affiliations:** Louisiana State University, Baton Rouge, Louisiana, United States; Louisiana State University, Baton Rouge, Louisiana, United States; Louisiana State University, Baton Rouge, Louisiana, United States; Louisiana State University, Baton Rouge, Louisiana, United States; Harvard University, Boston, Massachusetts, United States; Boston University, Boston, Massachusetts, United States

## Abstract

Exposure to multiple back-to-back disasters may threaten physical and mental health for people who live in geographic areas that are prone to severe weather events. In 2005, coastal residents from south Louisiana lost homes in Hurricane Katrina and many moved inland to presumably higher and safer ground. In August of 2016, historic flooding brought widespread destruction, creating a second round of catastrophic losses for those who had permanently relocated to Baton Rouge, Louisiana after Katrina. The present research is part of a larger longitudinal study on health and well-being after multiple disaster exposures. In this study, we examined associations among current and prior flood experience and health-related quality indexed by the SF-36 Health Survey (Ware & Sherbourne, 1992). Three flood exposure groups were compared across two waves of testing: non-flooded (controls), single disaster (flooded in 2016) and double disaster (flooded in 2005 and again in 2016). Results indicated that the flood exposure groups did not differ in physical health, although mental health was poorer for those whose homes flooded relative to the non-flooded controls at both waves of testing. Correlation analyses revealed that age was negatively associated with intolerance of uncertainty and physical health, but positively correlated with mental health at both waves of testing, consistent with the inoculation view of post-disaster psychological reactions. Implications of these data for understanding health-related quality of life after multiple disaster exposures are discussed.

